# Toward an Integrated Public Health Approach for Epilepsy in the 21st Century

**DOI:** 10.5888/pcd11.140270

**Published:** 2014-08-28

**Authors:** Howard K. Koh, Rosemarie Kobau, Vicky H. Whittemore, Marie Y. Mann, Jennifer G. Johnson, Joseph D. Hutter, Wanda K. Jones

**Affiliations:** Author Affiliations: Rosemarie Kobau, Vicky H. Whittemore, Marie Y. Mann, Jennifer G. Johnson, Joseph D. Hutter, Wanda K. Jones, US Department of Health and Human Services, Washington, DC.

## Abstract

Epilepsy, a complex spectrum of disorders, merits enhanced public health action. In 2012, the Institute of Medicine (IOM) released a seminal report on the public health dimensions of the epilepsies, recommending actions in 7 domains. The report urged a more integrated and coordinated national approach for care centering on the whole patient, including heightened attention to comorbidities and quality of life; more timely referral and access to treatments; and improved community resources, education, stakeholder collaboration, and public communication. The US Department of Health and Human Services responded to this report by accelerating and integrating ongoing initiatives and beginning new ones. This article summarizes recent federally supported activities promoting an integrated public health approach for epilepsy, highlighting progress in response to the landmark 2012 IOM report and identifying opportunities for continued public health action.

## Introduction and Background

Despite being recognized for millennia, epilepsy is poorly understood by the public today. Epilepsy represents a complex spectrum of disorders that varies by type, cause, severity, and impact (1,2). Epilepsy can not only shorten life but also severely compromise overall well-being and full participation in life activities (1–3). Hence, epilepsy is not just a trying neurological disorder for individuals but also a broader public health challenge for society (1,4–7). This article summarizes federally supported activities promoting an integrated public health approach for epilepsy, highlighting recent progress in response to the landmark 2012 Institute of Medicine (IOM) report, *Epilepsy Across the Spectrum: Promoting Health and Understanding* (2).

Some national efforts previously spurred planning to address epilepsy in the United States. For example, in 1975, the *Plan for Nationwide Action on Epilepsy,* commissioned by Congress and led by the former Department of Health, Education, and Welfare, identified the burden of epilepsy in the population and released a first set of groundbreaking recommendations to federal and state agencies (5). Later, 2 national conferences, Living Well with Epilepsy I (1997) and Living Well with Epilepsy II (2003), convened an expanded group of stakeholders to assess progress and gaps for continued public health action (7). Recommendations from these latter initiatives guided some federal agencies and epilepsy stakeholder activities in areas such as surveillance (2), but broader attention was needed.

To reinvigorate efforts to unify action in the 21st century, 24 organizations, including federal agencies in the US Department of Health and Human Services (HHS) and nonprofit organizations in Vision 20–20 (a group of epilepsy stakeholder organizations), joined together in 2010 to commission the first epilepsy report from the IOM. After receiving input through public meetings across the country and from health professional boards and associations, the IOM created a roadmap for the future. In March 2012, the IOM unveiled its seminal publication *Epilepsy Across the Spectrum: Promoting Health and Understanding* (2).

The report emphasized that epilepsy is a common spectrum of disorders that affects not only health but also quality of life for people of all ages; that a more coordinated approach is needed for care centering on the whole patient; that although effective treatments are available, timely referral and access to treatments fall short; and that it is an area filled with complexity and stigma. To address these and other challenges, the IOM recommended actions for a wide range of stakeholders in 7 domains (Table) (2).

**Table:** Selected Department of Health and Human Services (HHS) Activities Following the Release of the Institute of Medicine (IOM) Report *Epilepsy Across the Spectrum: Promoting Health and Understanding (2012)*

IOM Domain and Recommendations	Selected HHS Activities^a^
**Increasing the power of epilepsy data**	
1. Validate and implement standard definitions and criteria for epilepsy case ascertainment, health care and community services use and costs, and quality-of-life measurement. 2. Continue and expand collaborative surveillance and data collection efforts.	• Adoption of Common Data Element Project • Expansion of national, state, and local surveillance systems to examine epilepsy burden (eg, prevalence, access to care, comorbidity, early mortality) in the general population and high-risk groups • Development and tracking of a national objective on epilepsy in HHS *Healthy People 2020* to track access to care
**Preventing epilepsy**	
3. Develop and evaluate prevention efforts for epilepsy and its consequences.	• Identification of biomarkers of epileptogenesis • Identification of genetic factors that increase risk for epilepsy • Advancement of technologies to elucidate brain functioning for improved prevention and treatment opportunities • Examination of bioequivalence of generic and brand-name seizure medications • Development and testing of chronic disease self-management programs (secondary/tertiary prevention)
**Improving health care**	
4. Improve the early identification of epilepsy and its comorbid health conditions. 5. Develop and implement a national quality measurement and improvement strategy for epilepsy care. 6. Establish accreditation of epilepsy centers and an epilepsy care network. 7. Improve health professional education about the epilepsies.	• Elimination of pre-existing condition exclusions in health insurance policies and expanded coverage for care (eg, mental health care) via Affordable Care Act • Identification of promising practices of patient/family-centered coordinated, comprehensive care for medically underserved children and teens with epilepsy • Improvement of clinical services for adults with epilepsy and intellectual and developmental disabilities • Expansion of telehealth/telemedicine services to reach people living in rural and geographically isolated areas • Support of professional training opportunities for providers on epilepsy self-management
**Improving community resources and quality of life**	
8. Improve the delivery and coordination of community services.	• Implementation of evidence-based self-management programs for community-dwelling people with epilepsy • In partnership with the Epilepsy Foundation (national and state affiliates), provision of community-based services, education, and support to people with epilepsy and their caregivers
**Raising awareness and improving education**	
**Patient and family education** 9. Improve and expand educational opportunities for patients and families. **Public awareness and knowledge** 10. Inform media to improve awareness and eliminate stigma. 11. Coordinate public awareness efforts.	• Provision of free educational resources on epilepsy self-management programs and resources to people with epilepsy and their caregivers • Provision of professional training opportunities for school nurses, first responders, law enforcement, and other professionals to improve their understanding of epilepsy • In partnership with the national Epilepsy Foundation, implementation of national and targeted public awareness campaigns about epilepsy
**Strengthening stakeholder collaboration**	
12. Continue and expand Vision 20–20 working groups and collaborative partnerships.	• Collaboration in epilepsy stakeholder meetings, and other communities of practice • Expansion of partnerships with other clinical and public health stakeholders
**Engaging people with epilepsy and their families**	
13. Engage in education, dissemination of, and advocacy for improved epilepsy care and services.	—

a Activities are organized primarily by IOM domains, but some activities overlap more than 1 domain.

For HHS, the IOM report served as a catalyst for accelerating projects under way, launching new ones, and integrating departmental efforts (2,7). In 2 years since the report was released, HHS agencies have implemented numerous recommendations, often in collaboration with community partners and also by building on initiatives in the Affordable Care Act (ACA). This article documents these HHS efforts; of the 7 domains for which the IOM recommended actions, HHS has bolstered efforts in the first 5 domains, and in the remaining two has furthered partnerships with the voluntary, nongovernmental sector (through grants, contracts, or memoranda of understanding). The Table summarizes selected HHS activities, and we provide more information by domain below.

## Increasing the Power of Epilepsy Data

The IOM report urged validation and implementation of standard definitions and criteria throughout all aspects of epilepsy care, treatment, and prevention as well as expansion of data collection and surveillance efforts.

### Standard definitions

The National Institute of Neurologic Disorders and Stroke (NINDS) recently led the Common Data Element Project, which now standardizes the collection of epilepsy data by using common definitions and terminology throughout the National Institutes of Health (NIH) and for researchers nationwide. This standardized format should lead to common documentation, allow incorporation of epilepsy into clinical research on related conditions (such as traumatic brain injury and neurodevelopmental conditions), and facilitate data collection. Dissemination of this resource may foster universal adoption by epilepsy researchers and help them to reduce study start-up time, improve data quality, and promote data sharing across clinical research studies (8).

### Prevalence data and national goals

The IOM report stimulated new Centers for Disease Control and Prevention (CDC) efforts to clarify the burden of epilepsy in the United States. After the 2012 IOM report release, the CDC epilepsy program published updated figures on national prevalence (for the first time since 1994) using data from the 2010 National Health Interview Survey (NHIS), the flagship HHS survey that assesses some 42,000 households annually (9,10). Using validated questions for identifying clinical cases of epilepsy among community-dwelling adults, the analysis found that an estimated 4.1 million adults reported having epilepsy at some point in their lifetime (defined as being told by a physician or other health professional that they have epilepsy), while 2.3 million of them reported having active epilepsy (defined as having physician-diagnosed epilepsy and taking medications for seizures or having ≥1 seizures in the previous year) (9). The study documented that among adults with active epilepsy, only 52.8% had seen a neurologist or epilepsy specialist in the previous year, suggesting suboptimal care and problems with access (9).

A federal interagency workgroup used these NHIS epilepsy data to develop the first objective on epilepsy in *Healthy People*, the public health initiative that has set 10-year goals and objectives for the nation each decade since 1979. These new 2010 data meet *Healthy People 2020* objective criteria that ensure continuity and comparability of data over time and allow the country to set and track progress toward national targets for epilepsy into the 21st century (see “Improving Epilepsy Care” below) (11).

In addition to these national analyses, CDC has committed to expanding state, regional, and tribal (ie, American Indian communities) estimates of the burden of epilepsy (3,12,13). For example, the South Carolina Epidemiologic Study of Epilepsy is examining the burden of epilepsy (including rates of comorbidity, health care use, and early mortality) in that state (14). Investigators are identifying epilepsy cases with *International Classification of Diseases, 9th revision, Clinical Modification* (ICD-9-CM) codes (eg, 345.x) in a statewide administrative database that captures data on 2 million patient encounters for about 409,000 people with epilepsy (2000–2011). Using these data, a 2014 study found that about 57% of people with epilepsy (and only about 28% of controls) had both somatic and psychiatric/neurodevelopmental comorbidities (14).

### Comorbidities

By urging broader attention to documentation of epilepsy comorbidities, the IOM report supports the HHS Strategic Framework for Multiple Chronic Conditions, which emphasizes a patient-centered approach to care coordination (15). In this regard, CDC released a 2013 study of NHIS data (2010) to describe the burden of nonpsychiatric comorbidities in adults with epilepsy (16). Such adults were more likely than those without the condition to have 4 or more comorbidities (eg, cardiovascular disease, respiratory disorders) that reflect shared disease mechanisms, treatment side effects, and higher rates of risk factors (eg, cigarette smoking, physical inactivity) (16).

CDC also incorporated examination of epilepsy as a co-occurring condition into its existing cerebral palsy surveillance activities. Since its 2006 surveillance year, CDC has used its Autism and Developmental Disabilities Monitoring (ADDM) Network to track the co-occurrence of epilepsy among 8-year-old children with cerebral palsy in 4 US communities; 35% in 2006 and 41% in 2008 had co-occurring epilepsy (17,18). The methods built on a feasibility study at the Missouri ADDM Project that assessed the co-occurrence of epilepsy among children with cerebral palsy and autism spectrum disorder (ASD); 32.9% of children with cerebral palsy and 8.7% of children with ASD also had epilepsy (19).

### Epilepsy-associated mortality

Despite overall mortality rates among people with epilepsy 3 times higher than rates among people in the general population and sudden death risk 20 times higher (20), no US surveillance system had previously monitored sudden unexpected death in epilepsy (SUDEP). In 2013, with support from the NIH’s National Heart, Lung, and Blood Institute (NHLBI) and NINDS, CDC expanded its Sudden Unexpected Infant Death Case Registry to develop the new Sudden Death in the Young (SDY) Registry. The SDY registry will identify both SUDEP and sudden cardiac deaths among children and young adults aged 19 years or younger, consistent with child death review systems and protocols in most states. Funding in 2014 will support participation of up to 15 states or major metropolitan areas in the registry (21). Registry data could allow researchers to identify preventable risk factors or other mechanisms that may cause SUDEP.

Also, to expand surveillance of epilepsy-associated mortality, CDC is examining the National Vital Statistics System’s all-cause mortality files to identify possible cases of SUDEP among adults and also analyzing the National Violent Death Reporting System to clarify the rates of suicide among people with epilepsy.

## Preventing Epilepsy

In response to the IOM report’s call for more investigation that could lead to better primary, secondary, and tertiary prevention, HHS has moved toward more unified planning in that regard. For example, the Interagency Collaborative to Advance Research in Epilepsy (ICARE) joins NIH agencies, Vision 20–20, IOM, and key epilepsy stakeholder organizations, such as Citizens United for Research in Epilepsy (CURE), to promote research. Established several years ago, ICARE now uses the IOM report recommendations as a framework for its meetings, which bring more research attention to conditions beyond seizures that commonly affect epilepsy patients (including ASD, developmental and intellectual disabilities, cognitive impairment, and mental health conditions). In support of these efforts, NINDS has updated *2014 Benchmarks for Epilepsy Research* to encourage fuller exploration of the relationship of comorbidities with epilepsy and seizures, underlying disease mechanisms, and effects of epilepsy treatment (22).

NIH recently established the NIH NeuroBioBank, which coordinates human brain and tissue repositories. The NeuroBioBank website provides information about the donation process and about how postmortem tissue research can advance knowledge about brain disorders (23). President Obama’s new BRAIN (Brain Research through Advancing Innovative Neurotechnologies) initiative, which includes 6 new NIH funding opportunities, encourages the formation of interdisciplinary teams to develop new noninvasive imaging technologies for human research and advances technological capabilities for understanding how circuits of interacting neurons function to create behavior (24).

### Primary prevention and basic research

NIH-funded research could lead to improved prevention by identifying biomarkers of epileptogenesis in populations at risk (including those with tuberous sclerosis complex and traumatic brain injury and children with prolonged febrile seizures). The Epi4K multisite international collaboration funded by NINDS, which analyzes the genomes of more than 4,000 individuals with epilepsy and their families, recently identified new gene mutations associated with infantile spasms and Lennox–Gastaut syndrome (25), 2 severe forms of childhood-onset epilepsy associated with intellectual and developmental disabilities. Moreover, some recent research suggests congenital human papillomavirus infection could be a cause of epilepsy due to cortical dysplasia (26). Also, the National Institute of Child Health and Development’s Neonatal Research Network has helped develop standardized protocols involving head cooling in neonates with hypoxic-ischemic brain injury to reduce neurological impairments and mortality (27).

### Secondary and tertiary prevention: chronic disease self-management and drug and device interventions

The IOM report calls attention to epilepsy self-management (2), a theme reinforced by the ACA’s attention to patient chronic disease self-management (CDSM). CDSM entails improving knowledge and problem-solving skills in self-management so that patients can be full partners in their own care. In particular, the IOM report recognized research under way since 2007 by the CDC’s Prevention Research Centers’ Managing Epilepsy Well (MEW) Network (28).

Three self-management programs have been designed with the goals of improving self-management behaviors, reducing depression, improving quality of life, and eliminating barriers to care (eg, transportation, stigma, functional impairments) (28). WebEase (Epilepsy Awareness Support and Education), the first evidence-based online self-management program for adults with epilepsy (29), is available without cost on the national Epilepsy Foundation website (http://www.webease.org). Project UPLIFT (Using Practice and Learning to Increase Favorable Thoughts) and PEARLS (Program to Encourage Active Rewarding Lives) offer treatment for depression in adults with epilepsy, delivered by telephone, Internet, or home visits (30,31). UPLIFT and PEARLS intervention protocols are available at no cost to providers, but training for both programs is recommended. Since 2013, CDC has collaborated with the national Epilepsy Foundation to support evaluation of Project UPLIFT in Florida, Michigan, and New York. MINDSET (Management Information and Decision Support Epilepsy Tool) is a decision-support tool for use in clinics to improve patient–provider communication about self-management (32). Other MEW Network self-management programs under evaluation focus on enhancing social support and mood and cognitive management among adults and will report outcomes by late 2014.

Since the IOM report release, CDC has expanded its MEW Network from 4 to 6 university-based sites to evaluate self-management programs for adults with epilepsy and memory problems, adults with epilepsy and serious mental illness (eg, schizophrenia), and adults with epilepsy who live in rural areas. A request for proposals issued in March 2014 supports replication studies and cost-effectiveness studies of MEW Network programs and the development of new self-management programs in vulnerable groups (eg, children with epilepsy).

In the realm of devices and drugs for possible secondary and tertiary prevention, the Food and Drug Administration (FDA) announced in 2013 approval of a cranially implanted neurostimulator, which reduces seizure frequency (in people aged ≥18 years) with partial onset seizures refractory to 2 or more antiepileptic medications (33). FDA also has studies of drugs and devices relevant to epilepsy, including protocols examining bioequivalence of generic formulations of brand-name seizure medications in head-to-head comparisons.

## Improving Health Care

Although effective treatments are available for some people with epilepsy, too many lack access to specialist care, insurance coverage, care coordination, and quality care and treatment. The IOM report recommends earlier identification of epilepsy and its comorbidities, greater focus on quality of care, stronger epilepsy networks including epilepsy centers, and enhanced professional education.

### Access to specialist care and insurance coverage

Although many people with epilepsy may be treated effectively by primary care physicians, those with uncontrolled seizures require more specialized care provided by a neurologist or epilepsy specialist. People with uncontrolled seizures are at higher risk for adverse outcomes such as unemployment, driving limitations, loss of independence, and early mortality. A study of 13 states found that 34.9% of adults with active epilepsy and uncontrolled seizures in the previous 3 months did not see a neurologist or an epilepsy specialist in the previous 12 months, and about 20% of adults with epilepsy reported cost as a barrier to seeking care (3). On the basis of 2010 NHIS data, *Healthy People 2020* now includes as a national goal to increase the proportion of people with epilepsy and uncontrolled seizures (defined as people who reported being told by a doctor or health provider that they have epilepsy and reported ≥1 seizures of any type in the previous year) who receive appropriate care (ie, see a neurologist or epilepsy specialist at least once per year) from a baseline of 57.7% (in 2010) to 63.5% (in 2020) (11).The ACA prohibits insurance companies from refusing to sell or renew policies because of pre-existing conditions such as epilepsy (34). It also prohibits lifetime and annual dollar limits of health insurance coverage, thereby greatly reducing risks of medical bankruptcy for individuals. Improved insurance coverage could help improve access to specialist care. Because epilepsy is often a condition in children and young adults, it is noteworthy that at least 3.1 million young adults younger than 26 have obtained insurance coverage through their parents’ health plans since 2010 (35). Young adults have also accessed new coverage through Medicaid expansion and the ACA marketplaces.

Additionally, the provision of mental health coverage as part of essential health benefits made available through the ACA could also improve care for the large numbers of people with epilepsy and comorbid mental illness (2).

### Care coordination

The ACA authorizes demonstration projects to promote care coordination and team-based approaches to care delivery. In this spirit, the Health Resources and Services Administration’s (HRSA’s) Maternal and Child Health Bureau funds Project Access, which focuses on improving access to coordinated, comprehensive care for children and teens with epilepsy in medically underserved and rural areas. Consistent with IOM recommendations, these state and community-based demonstration projects, supported by a coordinating center at the American Academy of Pediatrics, promote patient/family–professional partnerships; link primary and specialty care, educational services, public health, and other community resources; focus on early detection and treatment; and address cultural and linguistic barriers to care.

### Quality of care

In its third round of funding in 2013, Project Access commits to quality measurement and the use of quality improvement methods (based on the Institute for Healthcare Improvement’s Breakthrough Series Model) to implement and validate a well-functioning community-based system of services for children and teens with special health care needs (36). As part of this process, the coordinating center and the new 2013 grantees will assess outcomes such as family satisfaction with professionals, levels of continuous coordinated care in a family-centered medical home, and whether families have adequate insurance to pay for services.

Expanding the evidence base on quality and documenting possible cost savings could lead to new payment models. As part of the ACA, the Centers for Medicare & Medicaid Services (CMS) enhanced support for care coordination in numerous areas. Also, a recent change in Medicaid rules allows for reimbursement for preventive services by qualified professionals established by the state if the service is recommended by a physician or other licensed practitioner (37). States have the authority to allow licensed practitioners to deliver care in nonclinical settings, such as in homes or schools. The CMS Innovation Center, created by the ACA, serves as a new locus to foster innovative care-delivery models seeking to improve quality and reduce costs.

### Epilepsy networks

The University Centers for Excellence in Developmental Disabilities (UCEDDs) (38), a national network funded by the HHS Administration for Community Living, supports individuals with intellectual and developmental disabilities, approximately 40% to 50% of whom have seizures. The Administration for Community Living funds 68 UCEDDs — at least one in every state and territory — to provide interdisciplinary training, community service, research, and information to increase independence, community integration, and inclusion of individuals with developmental and other disabilities (38). Each UCEDD develops its own priorities. Several, including UCEDDs in New York and Nebraska, have prioritized provision of clinical services for seizure control. Another, at the Westchester Institute for Human Development in New York, aims to reduce polypharmacy for epilepsy patients. Still others, such as the UCEDDs in Alaska, Washington, and Nevada, and at the University of Southern California, with HRSA funding have collaborated to improve outreach and access to care for individuals in rural areas.

### Telemedicine and telehealth to improve access

Some Project Access grantees and UCEDDs are using telemedicine and telehealth as a way to extend access to quality care and improve outcomes in rural and underserved areas. Telemedicine can overcome the physical distance between the care site and the health care expert to connect patients and their families, primary care practitioners, and epilepsy specialists in a patient-centered medical home, enhance real-time monitoring of test results and scans, and deliver health professional education. Early data from West Virginia, Michigan, and Nebraska indicate reduced wait times for neurology visits and less time and money spent by families traveling to specialist visits. In particular, the Nebraska UCEDD, as a Project Access grantee, was preliminarily able to reduce the wait time for telehealth consultation appointments for children; more formal evaluation of this program is needed. Also, the IOM report has spurred regional planning, including telecommunication technology options, in the 4-state region of Kansas, Nebraska, Iowa, and Missouri, which has almost 14 million people but only a few epileptologists.

### Professional education

Professional education can involve many types of providers, including primary care providers, school nurses, first responders, and law-enforcement personnel. These professionals can be trained to provide appropriate care and help link people with epilepsy to community supports. For example, the CDC-supported Epilepsy Foundation program, Managing Students with Seizures, trains school nurses about epilepsy and the needs of students with epilepsy. An evaluation of this program demonstrated increased confidence among nurses across numerous domains (eg, the ability to recognize partial seizures, knowing when to contact emergency help) (39). Another CDC-supported educational program implemented by Epilepsy Foundation affiliates across the country teaches first responders to identify the various symptoms associated with seizures, provide appropriate first aid response on scene, and determine situations warranting further medical care.

Similarly, CDC-supported law-enforcement training programs aim to improve recognition of seizures and awareness of the unique needs of people with epilepsy who may otherwise be subjected to physical restraint and be taken into police custody. Other CDC-supported training programs in local communities focus on child day care staff, adult day care staff, and middle- and high-school students and teachers. Initial evaluation results from these educational programs are promising, but additional studies are needed (6).

## Improving Community Resources and Quality of Life; Raising Awareness, and Improving Education

Beyond seizures, the impact of epilepsy involves challenges in schools, uncertainties about social and employment situations, limitations on driving, and questions about independent living. To address these needs, the IOM report urges improving community resources and improving education for people with epilepsy, their families, and the public.

### Patient and provider education

The CDC MEW Network provides free webinars and podcasts to patients, their caregivers, and health and social service providers about the benefits of epilepsy self-management. Since 2012, CDC has also supported provider training for UPLIFT (at no cost) and PEARLS with professional certification credits.

### Public education

CDC continues to partner with the Epilepsy Foundation to conduct public education and awareness campaigns (6). Although improvements in negative attitudes toward epilepsy have been reported over the decades (40), in 2002 about one-half of US adults believed that they were not knowledgeable about epilepsy, and only about 40% believed they knew what to do if someone had a seizure (2). In 2006, a study found that more than 80% of US community-dwelling adults disagreed with negative stereotypes of people with epilepsy (41), but substantial proportions of these adults expressed concerns about seizures, suggesting potential avoidance behaviors associated with perceptions of risk (41,42).

Epilepsy Foundation outreach campaigns that previously focused on African Americans and Hispanic Americans were extended in 2013 to Asian Americans. With combined English- and Chinese-language media in targeted US markets (Hawaii, Ohio, Oregon, Texas, Washington), the foundation’s Asian American outreach campaign reached almost an estimated 3 million people (P. Katana, oral communication, 2013). More formal evaluation is needed.

## Strengthening Stakeholder Collaboration and Engaging People With Epilepsy and Their Families

The last 2 domains from the IOM report highlight the critical roles of epilepsy stakeholders — including people with epilepsy and their families — in fostering and sustaining public health action (2). Organizing frameworks for stakeholder collaboration exist through various groups, including the Vision 20–20 working groups (2), the CDC MEW Network, the NINDS ICARE, Project Access, and the UCEDDs. Key groups such as CURE have advanced collaboration in vital ways. Participation of the broader public health community can support the IOM recommendations, elevate epilepsy as a public health issue, educate all about the new operational definitions for epilepsy (43), and potentially improve the lives of the millions of people living with epilepsy.

## Conclusion

The 21st century is the time for a more integrated public health approach to epilepsy. People with epilepsy need better coordinated care, social support, and treatments and greater social acceptance. The convergence of HHS and nonfederal partner interest has not only yielded a major public health roadmap through the 2012 IOM report but also accelerated the integration of activities through HHS agencies and nongovernmental partners.

Implementation of the IOM report recommendations stimulated activities to improve understanding of the burden of epilepsy, establish new surveillance systems for SUDEP, renew attention to comorbidities, accelerate new models for care coordination in the era of the ACA, explore improved outreach through telehealth, increase emphasis on improved access and quality of care, heighten collaboration on research for better prevention, and improve public and professional education for an array of stakeholders. Moreover, the country established targets for epilepsy as part of its *Healthy People 2020* process, which can monitor progress for the future.

Although the challenges ahead are considerable, these and future activities, in close collaboration with community-based partners and others, can bring renewed understanding of epilepsy as a public health priority. Continued commitment and momentum can move the country closer to a true integrated public health system for epilepsy for the 21st century.
